# PPM1D silencing by RNA interference inhibits the proliferation of lung cancer cells

**DOI:** 10.1186/1477-7819-12-258

**Published:** 2014-08-13

**Authors:** Chen Zhang, Yuanzhuo Chen, Mingsong Wang, Xianzhen Chen, Yongxin Li, E Song, Xiaoqing Liu, Sekwon Kim, Hu Peng

**Affiliations:** Shanghai Tenth People’s Hospital, Tongji University School of Medicine, Shanghai, 200072 China; Department of Cardiothoracic Surgery, Xinhua Hospital, Shanghai Jiaotong University School of Medicine, Shanghai, 200092 China; Department of Marine Bio Convergence Science, Specialized Graduate School Science and Technology Convergence, Pukyong National University, Busan, 608-737 Republic of Korea; Lixiang Eye Hospital of Soochow University, Suzhou, Jiangsu 215000 China

**Keywords:** PPM1D, lung cancer, shRNA, cell proliferation, cell cycle

## Abstract

**Background:**

PPM1D (protein phosphatase, Mg^2+^/Mn^2+^ dependent, 1D) has been reported to be involved in multiple human tumors. This study was designed to investigate the functional role of PPM1D in lung cancer cells.

**Methods:**

Expression levels of PPM1D were analyzed in A549 and H1299 cells by real-time PCR and Western blotting. Lentivirus-mediated short hairpin RNA (shRNA) was used to knock down PPM1D expression in both cell lines. The effects of PPM1D on lung cancer cell growth were investigated by MTT (3-(4,5-dimethylthiazol-2-yl)-2,5-diphenyltetrazolium bromide), colony formation and flow cytometry assays.

**Results:**

Knockdown of PPM1D in lung cancer cells resulted in decreased cell proliferation and impaired colony formation ability. Moreover, flow cytometry analysis showed that knockdown of PPM1D arrested cell cycle at the G_0_/G_1_ phase. Furthermore, PPM1D silencing downregulated the expression of cyclin B1 in H1299 cells. Therefore, it is reasonable to speculate that the mechanisms by which PPM1D knockdown alleviates cell growth may be partly via the induction of cell cycle arrest due to the suppression of cyclin B1.

**Conclusions:**

These results suggest that PPM1D silencing by RNA interference (RNAi) may be a potential therapeutic approach for the treatment of lung cancer.

## Background

Lung cancer is one of the major causes of death in the world [1]. The survival rate of lung cancer remains low despite the development of various treatment modalities [2, 3]. Even with advances in chemotherapy and radiotherapy, survival rates for patients with advanced stage disease remain largely unchanged [4]. The best chemotherapeutic agents have limited impact with median patient survival being only 11 to 13 months [5]. This raises the need for improved treatment methods based on molecular targeting of lung cancers [6]. The attention paid to understanding the molecular basis of carcinogenesis as a path for cancer defense is rapidly increasing [7, 8]. It has also been identified that targeting specific molecular phenomena of cancer development would be a more specific treatment approach. There are a large number of reports on the successful use of RNA interference (RNAi) to suppress cancer progression for both *in vitro* and *in vivo* models [9].

PPM1D (protein phosphatase, Mg^2+^/Mn^2+^ dependent 1D), also known as WIP1 (wild-type p53 induced protein phosphatase 1), is a member of the PP2C family of Ser/Thr protein phosphatases [10]. PPM1D transcription is upregulated in response to various types of DNA damage in a p53-dependent manner [11]. Once upregulated, PPM1D has been shown to dephosphorylate and downregulate several targets, particularly proteins associated with the ATM/ATR-initiated DNA damage response, including tumor suppressors with a proven role in cancer susceptibility such as p53 [12], ATM [13] and checkpoint kinase 2 (Chk2) [14]. There is also accumulating evidence that PPM1D is involved in oncogenesis. PPM1D amplification and overexpression have been demonstrated in multiple human tumors, including neuroblastoma [15], pancreatic adenocarcinoma [16], medulloblastoma [17], breast cancer [18, 19] and ovarian clear cell carcinoma [20]. For breast cancer, ovarian cancer, lung adenocarcinoma and hepatocellular carcinoma, PPM1D overexpression is associated with poor survival [21]. However, the functional role of PPM1D in lung cancer remains unclear.

Therefore, in this study, we examined the role of PPM1D in cell growth via an RNAi lentivirus system in two human lung cancer cell lines, A549 and H1299. The effects of PPM1D on lung cancer cell growth were investigated by MTT (3-(4,5-dimethylthiazol-2-yl)-2,5-diphenyltetrazolium bromide), colony formation and flow cytometry assays.

## Methods

### Reagents and plasmids

Dulbecco’s modified Eagle’s medium (DMEM), RPMI1640 medium and fetal bovine serum (FBS) were obtained from Hyclone (Logan, UT, USA). Short hairpin RNA (shRNA) expression vector pFH-L, lentiviral packaging aid vectors pVSVG-I and pCMVΔR8.92 were purchased from Shanghai Hollybio (Shanghai, China). RNeasy MidiKit was purchased from Qiagen (Valencia, CA, USA). AgeI, EcoRI, and SYBR Green Master Mix Kits were purchased from TaKaRa (Dalian, China). Lipofectamine 2000 and TRIzol were purchased from Invitrogen (Carlsbad, CA, USA). M-MLV reverse transcriptase was purchased from Promega (Madison, WI, USA). All other chemicals were obtained from Sigma (St Louis, MO, USA). The antibodies used were as follows: anti-PPM1D (1:500 dilution; Abcam, Cambridge, UK), anti-GAPDH (1:5,000 dilution; Santa Cruz, CA, USA), anti-mouse HRP and anti-rabbit HRP (1:5,000 dilution; Santa Cruz).

### Cell culture

Human lung cancer cell lines, A549 and H1299, and human embryonic kidney cell line 293 T were obtained from the cell bank of the Shanghai Institute of Cell Biology. A549 and 293 T cells were maintained in DMEM supplemented with 10% heat-inactivated FBS and penicillin/streptomycin. H1299 cells were maintained in RPMI1640 medium supplemented with 10% heat-inactivated FBS and penicillin/streptomycin. All cells were incubated at 37°C in a humidified atmosphere containing 5% CO_2_.

### Construction of PPM1D short hairpin RNA containing lentivirus and transduction into lung cancer cells

To construct lentiviruses containing PPM1D shRNA and control non-silencing shRNA (shCon), the siRNA sequences 5′-CCCTTCTCGTGTTTGCTTAAA-3′ and 5′-TTCTCCGAACGTGTCACGT-3′ were used, respectively. These nucleotide sequences were inserted into the plasmids using a vector expressing pFH-L shRNA. Lentiviruses were generated by triple transfection of 80% confluent 293 T cells with modified pFH-L plasmid and pVSVG-I and pCMV△R8.92 helper plasmids using Lipofectamine 2000. Then the lentiviral particles were harvested by ultra-centrifugation (4,000 *g* at 4°C) for 10 min, filtered through a 45-μm filter, and centrifuged (4,000 *g* at 4°C) again for 15 min.

For cell infection, A549 and H1299 cells were cultured in six-well plates at a density of 5 × 10^5^ cells per well and transduced with the constructed lentiviruses containing PPM1D shRNA (Lv-shPPM1D) and non-silencing shRNA (Lv-shCon) at an MOI of 35 and 20, respectively. The infection efficiency was measured after 72 h through a fluorescence microscope by observing the expression of green fluorescent protein.

### RNA extraction and real-time PCR analysis

Total RNA was extracted from cells using TRIzol reagent and synthesized into cDNA by M-MLV reverse transcriptase according to the manufacturer’s instructions. Real-time quantitative PCR was performed on a BioRad Connect Real-Time PCR platform using SYBR Green Master Mix Kit. In brief, each PCR reaction mixture containing 10 μl of 2 × SYBR premix ex taq, 0.8 μl of sense and antisense primers (2.5 μM), 5 μl of cDNA and 4.2 μl of ddH_2_O, was run for 40 cycles with initial denaturation at 95°C for 1 min, denaturation at 95°C for 5 s and extension at 60°C for 20 s. The forward and reverse primers of PPM1D were 5′-AGAGAATGTCCAAGGTGTAGTC-3′ and 5′-TCGTCTATGCTTCTTCATCAGG-3′. β-actin was used as an internal control. The forward and reverse primers of β-actin were 5′-GTGGACATCCGCAAAGAC-3′ and 5′-AAAGGGTGTAACGCAACTA-3′. Relative gene expression levels were calculated using 2^-ΔΔCT^ analysis.

### Western blot analysis

Cells were collected 6 days after lentivirus infection and lysed in a radio-immune precipitation assay buffer. The protein content was measured by the Lowry method. Each protein sample was adjusted to 2 μg/μl in 20 μl volume mixed with 2× SDS sample buffer, electrophoresed on a 12% SDS-PAGE gel and transferred to a polyvinylidene difluoride membrane. The protein levels were detected after antibody treatment using ECL kit (Amersham, Piscataway, NJ, USA) by exposure to X-ray film.

### MTT assay

Lentivirus-transduced cells were seeded into 96-well plates at a concentration of 2 × 10^3^ cells per well. Following incubation for 1, 2, 3, 4 or 5 days, 20 μl of MTT (5.0 mg/ml) was added to each well. Following incubation at 37°C for 4 h, 200 μl of dimethyl sulfoxide was added to each well after removing the medium and MTT from the wells. The absorbance was measured using a micro-plate reader at 595 nm.

### Colony formation assay

Lentivirus-transduced cells were seeded into six-well plates at a concentration of 200 cells per well. The medium was refreshed every 3 days. After 8 days of culturing, the cells were washed with PBS and fixed with 4% paraformaldehyde. The fixed cells were stained with freshly prepared Giemsa stain for 20 min. Colonies were counted under light/fluorescence microscopy.

### Cell cycle analysis

The cell cycle distribution was analyzed by flow cytometry using propidium iodide staining. After lentivirus infection for 4 days, H1299 cells were seeded on six-well plates at a density of 5 × 10^5^ cells per well. Then the cells were collected at 80% confluency, fixed by suspending in 0.7 ml of 70% cold ethanol and incubated for 30 min at 4°C. The ethanol was discarded by centrifugation and the propidium iodide (100 μg/ml) solution containing 10 μg/ml of DNase-free RNase A was added and incubated for 30 min. Then the cell suspension was filtered through a 50-μm nylon mesh, and the stained cells were analyzed by flow cytometer (FACS Cali-bur, BD Biosciences, San Diego, CA, USA).

### Statistical analysis

All data are presented as mean ± standard deviation (SD) of at least three independent experiments performed in triplicate. The statistical analysis used Student’s *t*-test and *P* < 0.05 was considered to be statistically significant.

### Ethical approval statement

All experimental research that is reported in this manuscript has been performed with the approval of the Institutional Ethics Committee of Tongji University.

## Results

### Effect of infection by lentiviruses containing PPM1D short hairpin RNA on PPM1D expression in lung cancer cells

**Figure 1:**
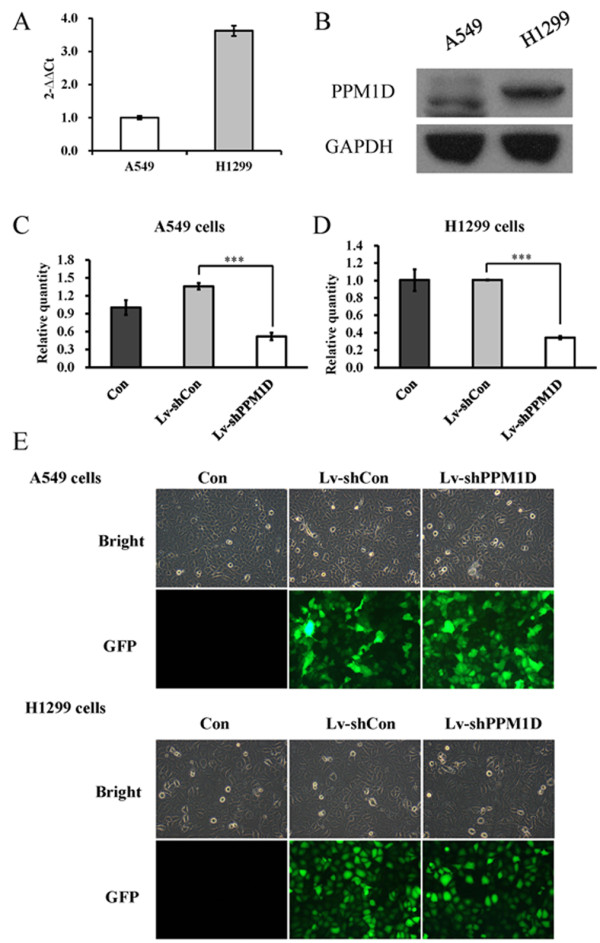
**Expression levels of PPM1D in lung cancer cells. (A)** Gene expression levels of PPM1D in A549 and H1299 cells analyzed by real-time PCR. **(B)** Protein expression levels of PPM1D in A549 and H1299 cells analyzed by Western blotting. **(C)** PPM1D gene expression levels in Lv-shPPM1D infected, Lv-shCon infected and non-infected (Con) A549 cells. **(D)** PPM1D gene expression levels in Lv-shPPM1D infected, Lv-shCon infected and non-infected H1299 cells. **(E)** Light microscopic and florescence microscopic pictures of Lv-shPPM1D infected, Lv-shCon infected and non-infected A549 and H1299 cells. Values are the mean of three independent experiments; bars represent SD. ***P < 0.001 in comparison with control. Con, control; GFP, green fluorescent protein.

To explore the role of PPM1D in lung cancer, we first detected the expression levels of PPM1D by real-time PCR and Western blotting in two lung cancer cell lines, A549 and H1299. As shown in Figure 1A, B, both cell lines had PPM1D mRNA and protein expression. However, the expression level of PPM1D in H1299 cells was higher than that in A549 cells. To examine the function of PPM1D in lung cancer cells, we used lentivirus-mediated shRNA to knock down PPM1D expression in both A549 and H1299 cells. As shown in Figure 1C, D, the relative expression levels of PPM1D were significantly (*P* < 0.001) reduced in Lv-shPPM1D infected cells, compared to non-infected cells and Lv-shCon infected cells. The knockdown efficiency of PPM1D was calculated as 61.9% for A549 cells and 65.8% for H1299 cells. Moreover, more than 90% of cells expressed the green fluorescence protein, indicating that lentivirus infection was successful (Figure 1E). There results indicate that the lentiviruses containing PPM1D shRNA could efficiently suppress the expression of endogenous PPM1D in lung cancer cells.

### Effect of PPM1D short hairpin RNA on the viability of lung cancer cells

**Figure 2:**
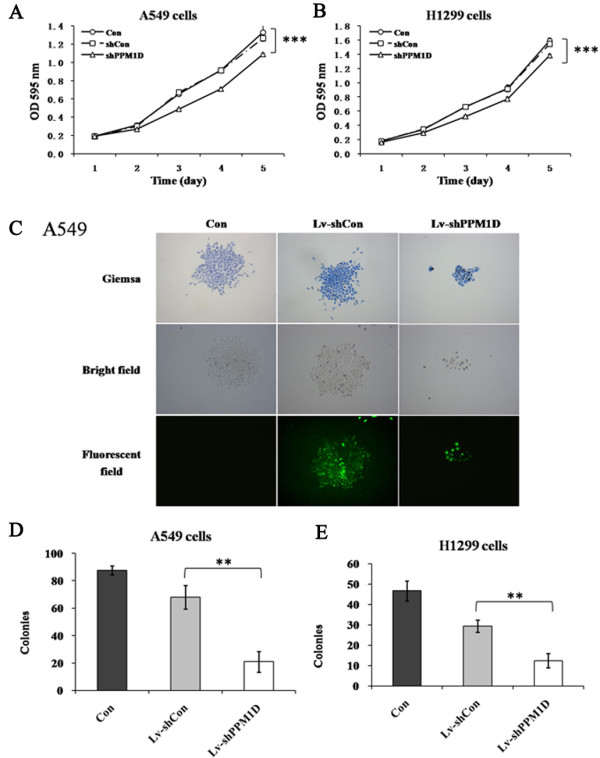
**Effect of siRNA-mediated PPM1D knockdown on the proliferation of lung cancer cells.** A549 **(A)** and H1299 **(B)** cells analyzed using the MTT assay. **(C)**. Light microscopic and fluorescence microscopic pictures of the colonies of A549 cells. Colonies were stained with Giemsa staining after 8 days of infection. Number of colonies of Lv-shPPM1D infected, Lv-shCon infected and non-infected A549 **(D)** and H1299 **(E)** cells. Values are the mean of three independent experiments; bars represent SD. **P < 0.01 and ***P < 0.001 in comparison with the control. Con, control; OD, optical density.

To explore the effect of PPM1D silencing on the viability of lung cancer cells, an MTT assay was performed for A549 and H1299 cells. Cell viability was observed for 5 days for non-infected cells, Lv-shCon infected cells and Lv-shPPM1D infected cells. As depicted in Figure 2A, B, the growth curve of Lv-shPPM1D infected cells started to drop from the third day, compared to non-infected cells and Lv-shCon infected cells. On the fifth day, the difference for cell viability was significantly wider (*P* < 0.01), while there was no difference between non-infected cells and Lv-shCon infected cells. These results indicate that knockdown of PPM1D could remarkably inhibit the viability of lung cancer cells.

### Effect of PPM1D short hairpin RNA on the colony-forming ability of lung cancer cells

To explore the long-term effect of PPM1D silencing on the proliferation of lung cancer cells, an assay of colony formation was performed for A549 and H1299 cells. As shown in Figure 2C, the size of each single colony of Lv-shPPM1D infected cells was much smaller than for non-infected cells or Lv-shCon infected cells. Moreover, the number of colonies was significantly decreased (*P* < 0.01) for both A549 and H1299 cells after PPM1D silencing (Figure 2D, E). These results indicate that knockdown of PPM1D could remarkably inhibit the proliferation of lung cancer cells.

### Effect of PPM1D silencing on the cell cycle distribution of H1299 cells

**Figure 3:**
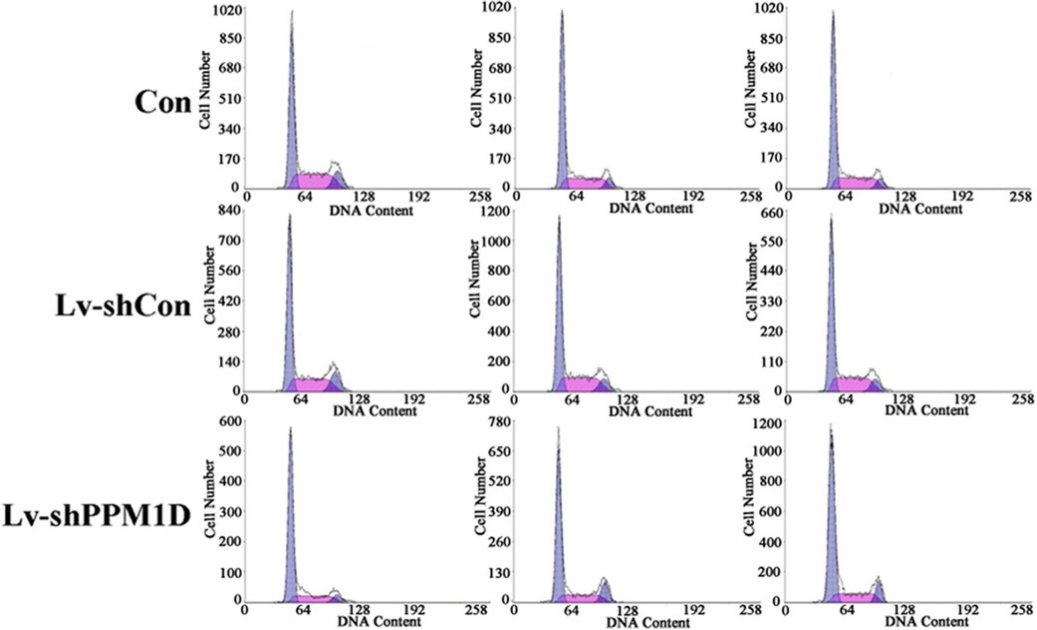
**Fluorescence-activated cell sorting histograms showing cell cycle distributions of Lv-shPPM1D infected, Lv-shCon infected and non-infected H1299 cells.** Con, control. Each line is the repeats form three independent experiments.

**Figure 4:**
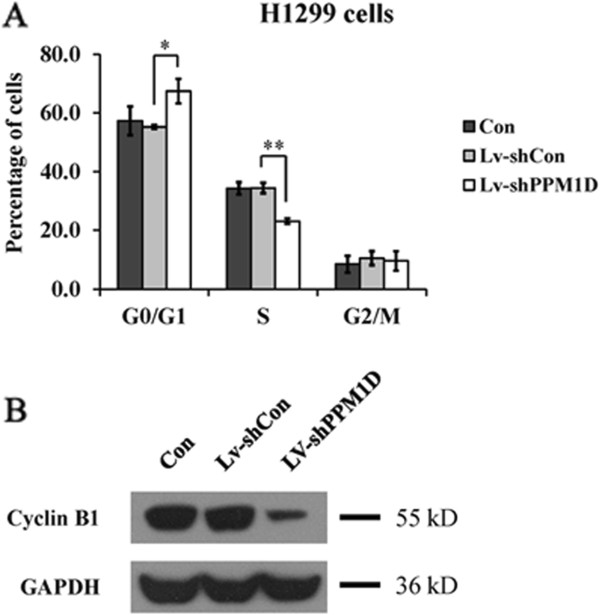
**Effect of PPM1D knockdown on the cell cycle distribution of H1299 cells. (A)** Percentage of cells residing in each checkpoint of the cell cycle. **(B)** Cyclin B1 protein expression levels of Lv-shPPM1D infected, Lv-shCon infected and non-infected H1299 cells analyzed by Western blotting. Values are the mean of three independent experiments; bars represent SD. *P < 0.05 and **P < 0.01 in comparison with the control. Con, control.

The effect of PPM1D silencing on cell cycle distribution was analyzed using a flow cytometer. As shown in Figures 3 and 4A, PPM1D knockdown seriously affected the cell cycle distribution of H1299 cells. The cell percentage for the G_0_/G_1_ phase was significantly increased (*P* < 0.05) for Lv-shPPM1D infected cells compared to non-infected cells and Lv-shCon infected cells. Meanwhile, the cell population of the S phase showed a marked decrease (*P* < 0.01) of H1299 cells after PPM1D silencing. These results indicate that knockdown of PPM1D could arrest the cell cycle at the G_0_/G_1_ phase. Furthermore, the expression level of mitosis-related protein cyclin B1 was obviously reduced in H1299 cells after PPM1D silencing (Figure 4B). Taken together, we suggest that knockdown of PPM1D can suppress lung cancer cell growth via a blockade of cell cycle progression and mitosis.

## Discussion

Molecular targeted therapies are now included in the treatment regimen for lung cancer since they have been shown to extend progression-free survival and improve overall survival [22–24]. PPM1D was first identified in a screen for p53 target genes induced by ionizing radiation [25], and has been proposed as a homeostatic regulator of the DNA damage response, facilitating the return of cells to their normal state after the repair of damaged DNA [12]. PPM1D could inhibit p53 signaling, and is putatively oncogenic [26]. In addition to p53 inhibition, PPM1D downregulates p38 mitogen-activated protein kinase [27, 28]. To date, there is accumulating evidence that PPM1D is involved in multiple human tumors, including neuroblastoma, pancreatic adenocarcinoma, medulloblastoma, breast cancer, ovarian cancer and hepatocellular carcinoma, and is a promising therapeutic target [29].

Gene knockdown using RNAi is an excellent tool for assessing the functional importance of cancer related genes *in vitro*. PPM1D silencing using lentivirus-mediated RNAi has been identified as a potential therapeutic approach for the treatment of human glioma [30]. Recently, oncogenic PPM1D has been identified as a novel prognostic marker for the survival of patients with lung cancer [31]. In the present study, we found that PPM1D is expressed in two human lung cancer cell lines, A549 and H1299. Therefore, to investigate the functional role of PPM1D in lung cancer, we employed lentivirus-mediated shRNA to knock down PPM1D expression in both cell lines. Knockdown of PPM1D in lung cancer cells resulted in decreased cell proliferation and impaired colony formation ability, which are in line with a previous report showing that downregulation of PPM1D by RNAi inhibited proliferation of glioma cells.

PPM1D plays a crucial role in the DNA damage response by inhibiting several cell cycle proteins, including p53. To elucidate the underlying mechanism of cell growth inhibition, cell cycle progression was then analyzed for H1299 cells after PPM1D silencing. Flow cytometry analysis showed that knockdown of PPM1D arrested the cell cycle at the G_0_/G_1_ phase. Moreover, PPM1D silencing downregulated the expression of cyclin B1 in H1299 cells. Therefore, it is reasonable to speculate that the mechanisms by which PPM1D knockdown alleviates cell growth may be partly via the induction of cell cycle arrest due to the suppression of cyclin B1. A previous study of PPM1D null mice showed that PPM1D exhibits oncogenic activity *in vitro* and *in vivo*, and the oncogenic function of PPM1D is associated with its phosphatase activity [11]. Further investigations should be conducted to unravel the regulatory mechanism of oncogenic phosphatase PPM1D in lung cancer cells.

Collectively, our results demonstrate that PPM1D is a key player in lung cancer cell growth. This study reveals a potential therapeutic approach based on targeting PPM1D and further *in vivo* studies are planned to confirm whether it is a potent target for lung cancer treatment.

## Conclusions

This study provides evidence for the first time that PPM1D modulates lung cancer cell proliferation via cell cycle control. PPM1D silencing by RNAi may be a potential therapeutic approach for the treatment of lung cancer.

## Abbreviations

DMEM: (Dulbecco’s) modified Eagle’s medium
FBS: fetal bovine serum
Lv-shCon: lentiviruses containing non-silencing shRNA
Lv-shPPM1D: lentiviruses containing PPM1D shRNA
MTT: 3-(4,5-dimethylthiazol-2-yl)-2,5-diphenyltetrazolium bromide
PBS: phosphate-buffered saline
PCR: polymerase chain reaction
PPM1D: protein phosphatase Mg^2+^/Mn^2+^ dependent, 1D
RNAi: RNA interference
SD: standard deviation
shCon: control non-silencing shRNA
shRNA: short hairpin RNA
siRNA: small interfering RNA
WIP1: wild-type p53 induced protein phosphatase 1.
